# Impact of Mobile Oral Health Applications on Pediatric Dental Anxiety: A Systematic Review and Network Meta-Analysis

**DOI:** 10.7759/cureus.106756

**Published:** 2026-04-09

**Authors:** Shanthosh Raj Srinivasan, Ravi Karthikayan, KC Vignesh, Bala Chaithanya Prasad Kota

**Affiliations:** 1 Dentistry, Employees' State Insurance Corporation (ESIC) Medical College and Hospital, KK Nagar, Chennai, IND; 2 Public Health Dentistry, Ragas Dental College and Hospital, Chennai, IND; 3 Pediatric and Preventive Dentistry, Sri Ramachandra Dental College and Hospital, Sri Ramachandra Institute of Higher Education and Research, Chennai, IND; 4 Pediatric and Preventive Dentistry, Sri Venkateswara Dental College and Hospital, Chennai, IND

**Keywords:** behaviour management techniques, health interventions, mobile oral health application, pediatric dental anxiety, tell-show-do technique

## Abstract

Dental anxiety (DA) in children remains a significant barrier to effective oral healthcare, often leading to treatment avoidance and poor outcomes. While traditional behavioral techniques such as Tell-Show-Do (TSD) and animated videos have been widely used, emerging digital interventions, particularly mobile oral health applications, offer novel, child-friendly approaches for anxiety reduction. Thus, the aim of this review is to evaluate the effectiveness of mobile oral health applications in reducing DA among 4-11-year-olds, compared to other non-pharmacological behavioral management techniques. This review included 13 randomized controlled trials (RCTs) involving 1,002 children aged 4-11 years. Ten studies were eligible for meta-analysis. Information sources included PubMed, Cochrane Library, CINAHL, ScienceDirect, and Google Scholar, with the last search conducted on June 15, 2025. Interventions included mobile applications, maternal voice recordings, animated videos, and chairside techniques (TSD, TPD (Tell-Play-Do), Tell-Show-Play-Distraction (TSPD)). Primary outcomes were changes in validated anxiety scales; secondary outcomes included heart rate and oxygen saturation. Risk of bias was assessed using the Cochrane Risk of Bias 2 (ROB 2) tool for randomized trials and the ROBINS-I tool for non-randomized studies. A Bayesian hierarchical model was applied to rank interventions using surface under the cumulative ranking curve (SUCRA) probabilities. Mobile applications significantly reduced anxiety scores and heart rate compared to the TSD and control groups. TSPD ranked highest for anxiety reduction (SUCRA: 99.53%), followed by maternal voice (66.34%) and mobile apps (60.23%). For heart rate reduction, maternal voice (83.24%) and mobile apps (76.5%) were most effective. Funnel plot asymmetry and Egger’s test indicated potential publication bias. Mobile oral health applications, particularly when combined with maternal voice or presence, are effective and scalable complements to traditional behavioral techniques for managing pediatric DA. However, these findings should be interpreted with caution, as the network meta-analysis demonstrated moderate heterogeneity across included studies.

## Introduction and background

The first dental visit is often marked by fear of the unknown, which can subsequently develop into anxiety. Dental anxiety (DA), originally defined as "the patient’s response to stress specific to a dental situation" [1], remains a significant challenge in oral healthcare. It represents a cognitively involved emotional reaction to dental stimuli or experiences [2], characterized by both physical and psychological responses to perceived threats.

In children, DA presents a significant challenge in pediatric dentistry, with reported prevalence rates ranging from 5.7% to 19.5% [3] and an estimated average of around 9% [4]. The development of DA in children is influenced by multiple factors, with strong associations observed between parental and child anxiety [5]. These observations have prompted the development of various behavior guidance techniques aimed at managing anxiety and disruptive behavior in pediatric dental patients.

Traditionally, DA in children is managed through pharmacological and non-pharmacological approaches, with the latter generally preferred for their safety, cost-effectiveness, and efficacy. According to the American Academy of Pediatric Dentistry (AAPD), basic behavior guidance techniques are grounded in education, communication (e.g., counseling skills to establish rapport and trust, non-verbal communication), and fundamental psychological and behavioral principles (e.g., direct observation, positive reinforcement, and distraction). Contemporary pediatric dentistry increasingly emphasizes child-centered psychological behavioral therapy, such as pretend play, audio-visual aids, virtual reality, and animal-assisted therapy. Distraction techniques are aimed at minimizing anxiety, reducing unpleasantness, and preventing negative or avoidance behaviors during dental treatment [6].

In alignment with current AAPD recommendations, the term "behavior guidance techniques" is used in place of the older term "behavior management techniques." Behavior guidance is proactive and teaches self-regulation, while behavior management is reactive and controls behavior through rules and consequences. This shift in terminology reflects a child-focused approach in helping pediatric patients cope with dental procedures [6].

Behavior guidance techniques employed two decades ago have evolved substantially in response to advancements in clinical practice, and many of these earlier approaches may no longer accurately represent contemporary pediatric dental care. Moreover, the AAPD best practice guidelines have undergone multiple revisions since their initial adoption in 1990, further limiting the relevance and applicability of conclusions derived from earlier studies [6].

Recent technological advancements have expanded the scope of behavior guidance through digital interventions. Children interact with digital dental content via engaging visual and interactive media, while caregivers use these platforms to access and evaluate information. As primary decision-makers, caregivers, particularly mothers, play a crucial role in shaping the impact of such interventions on dental treatment outcomes [7]. Mobile-based, pedodontist-approved dental game applications have demonstrated potential in reducing dental fear by familiarizing children with dental procedures prior to treatment via parent-owned smartphones [8]. These applications simulate common dental procedures, such as tooth brushing, dental cleaning, teeth filling, and extractions, using graded audiovisual stimuli to enhance acceptance and cooperation [9].

In pediatric dentistry, child-friendly mobile dental applications use gamification to familiarize children with dental environments. A commercial game (Crazy Dentist - Fun Games 1.0) was shown to reduce heart rate and anxiety during anesthesia and drilling at first dental appointments [8]. Similarly, the Tell-Play-Do (TPD) approach is designed to reduce child anxiety, which involves explaining the procedure (Tell), letting the child use toy instruments on a model (Play), and then doing the procedure (Do). Combining the TPD approach with mobile apps effectively lowered pediatric dental fear, especially during procedures involving high-speed dental drills [10].

Despite these encouraging outcomes, most commercially available apps are not specifically designed to address pediatric DA. Nevertheless, when integrated with established behavioral strategies, mobile health applications represent a scalable, engaging, and child-friendly adjunct for the management of DA in children.

A recent systematic review evaluated behavioral techniques used during preventive dental visits, including modeling and mobile applications, but excluded advanced approaches such as cognitive behavioral therapy (CBT) and virtual reality (VR) [11,12]. Additional systematic reviews have analyzed modern psychological methods [13-15], with some focusing specifically on VR, video modeling [16,17], and music as distraction techniques [18-20].

Mobile apps can reduce anxiety by gradually exposing users to feared situations (desensitization), increasing comfort through repeated use (familiarity), diverting attention away from stress (distraction), and demonstrating positive coping behaviors (modeling).

Most existing research on digital interventions for pediatric DA has concentrated on immersive technologies such as virtual reality. While VR has demonstrated efficacy, its limited accessibility and requirement for specialized equipment restrict widespread use in routine pediatric dental practice. In contrast, mobile applications have become an integral part of daily life, offering scalable, accessible, and child-friendly platforms that can be easily integrated into dental settings. Despite encouraging findings from individual studies, evidence on mobile oral health applications remains fragmented. There is no clear consensus on the most effective app features or approaches. A network meta-analysis enables simultaneous comparison of multiple interventions, providing a comprehensive ranking of effectiveness to guide clinical decision-making. Therefore, this systematic review and network meta-analysis aim to comprehensively evaluate and compare the effectiveness of mobile oral health applications with other non-pharmacological behavior guidance techniques in reducing DA among pediatric patients.

## Review

Methodology

Study Design and Registration

This network meta-analysis was conducted in alignment with the updated PRISMA (Preferred Reporting Items for Systematic reviews and Meta-Analyses) 2020 guidelines for systematic reviews and network meta-analyses [21]. The study protocol was prospectively registered in the PROSPERO database (Registration ID: CRD420251139593). Study selection was guided by the PICOS (Population/Problem, Intervention, Comparison, Outcome, and Study Design) framework as outlined in Table 1.

**Table 1:** Research question and eligibility criteria for the study

Research question: What is the effect of mobile oral health applications compared to other non-pharmacological behavioral management techniques on reducing anxiety levels in pediatric patients during dental procedures?		
PICOS	Inclusion	Exclusion
Population	Pediatric patients aged 2-14 years undergoing dental procedures with anxiety	Children under the age of 2 and adolescents above 14 years, and studies on special children or with developmental disabilities, with anxiety, and dental procedures carried out in children without anxiety
Intervention	Use of mobile oral health applications (Android/iOS) that provide interactive or instructional content designed for children (Educational apps, Game apps)	Mobile apps not specifically designed for dental health or anxiety management
Comparator	Standard dental care (no use of mobile applications) or other behavioral management interventions for managing dental anxiety	Studies lacking a comparator group
Outcomes	Primary outcomes: reduction in pediatric dental anxiety levels, measured by validated anxiety scales. Secondary outcomes: heart rate	Studies reporting only qualitative outcomes or not reporting any outcomes
Study design	Randomized controlled trials, clinical trials	Case reports, case series, narrative reviews, expert opinions, editorials, letters, conference abstracts, meta-analysis, animal studies, or in vitro experiments

Literature Search

A systematic search was performed to identify randomized controlled trials (RCTs) across five electronic databases: PubMed, Cochrane Library, CINAHL, ScienceDirect, and Google Scholar. The search strategy incorporated Boolean operators and relevant keywords, including (smartphone dental application OR mobile health OR mobile application OR e-health OR m-health OR Little Lovely Dentist OR oral health mobile application) AND (dental anxiety) AND (pediatric dentistry OR children). Two authors independently executed the search and screened the results. No restrictions were applied regarding language or publication date. The literature search was conducted from inception up to June 15, 2025. Details of the search strategy are presented in Table 2.

**Table 2:** Search strategy for the study

Database	Search string	N
PubMed	((smartphone dental application OR mobile health OR mobile application OR smartphone application OR e-health OR m-health OR Little lovely dentist OR oral health mobile application) AND (Dental anxiety)) AND (pediatric dentistry or pediatrics OR children)	34
Cochrane library	((smartphone dental application OR mobile health OR mobile application OR smartphone application OR e-health OR m-health OR Little lovely dentist OR oral health mobile application) AND (Dental anxiety)) AND (pediatric dentistry or pediatrics OR children)	21
CINAHL	((smartphone dental application OR mobile application OR Little Lovely Dentist OR oral health mobile application) AND (dental anxiety)) AND (pediatric dentistry or pediatrics OR children)	19
ScienceDirect	((smartphone dental application OR mobile application OR Little Lovely Dentist OR oral health mobile application) AND (dental anxiety)) AND (pediatric dentistry or pediatrics OR children)	276
Google Scholar	((“smart phone dental application” OR “mobile health” OR “mobile application” OR “smart phone application” OR “e-health” OR “m-health” OR “Little lovely dentist” OR “oral health mobile application”) AND (“Dental anxiety”)) AND (“pediatric dentistry” or “Pediatrics” OR “children”)	164

Screening

All identified records were systematically managed using Zotero reference software and subsequently assessed for eligibility via the Rayyan platform (Rayyan Systems Inc., Cambridge, United States; https://rayyan.ai/). Duplicate entries were detected and excluded within Rayyan before initiating screening. Title and abstract screening were carried out independently by two reviewers (SR and K). Inter‑rater agreement for this stage was strong (Cohen’s Kappa κ = 0.89). Full‑text articles were then assessed independently by the same reviewers, with strong agreement (Cohen’s Kappa κ = 0.85). Discrepancies at any stage were resolved through discussion or consultation with a third reviewer (VK). For articles without accessible full texts, efforts were made to retrieve them by contacting the corresponding authors or journal editorial teams.

Data Extraction

Data extraction was performed independently by two reviewers (SR and K) using a standardized sheet developed a priori in Microsoft Excel (Microsoft Corporation, Redmond, Washington). The sheet captured study characteristics (author, year, country, design), participant demographics, intervention details, comparator types, outcome metrics, and risk-of-bias domains. The primary outcome of interest was the change in DA scores, assessed through validated scales at post‑intervention. Secondary outcomes included physiological and behavioral measures such as heart rate, pain intensity, and oxygen saturation. Inter‑rater agreement for extracted variables was high (Cohen’s Kappa κ = 0.87), indicating strong consistency between reviewers. Discrepancies were resolved through discussion with a third reviewer (VK) to ensure accuracy.

Quality Assessment

The methodological quality of the included RCTs was evaluated using the Cochrane Risk of Bias 2.0 (RoB2) tool [22]. Five domains were assessed: randomization process, deviations from intended interventions, missing outcome data, measurement of the outcome, and selection of the reported result. Non‑randomized trials were evaluated using the ROBINS‑I tool [23], which addresses seven domains of bias. Each study was independently appraised by two reviewers, with judgments categorized as "low risk," "some concerns," or "high risk" for each domain. Any discrepancies between reviewers were resolved through discussion or, when necessary, consultation with a third reviewer. The overall risk of bias profile was visualized using a summary graph generated through the RoB2 (robvis visualization) tool interface [24].

Statistical Analysis

This network meta-analysis focused on two primary outcome measures: DA and heart rate. Anxiety reduction was quantified using standardized mean differences (SMDs) with 95% CIs, while heart rate changes were expressed as mean differences (MDs) with corresponding 95% CIs. Due to the heterogeneity in anxiety scales and measurement units across studies, SMD was selected to standardize effect sizes and facilitate pooled analysis, in accordance with the Cochrane Handbook for Systematic Reviews of Interventions.

To compare multiple interventions simultaneously, a Bayesian hierarchical random-effects model was employed using the MetaInsight platform (v.6.4.0), which interfaces with JAGS through R (v4.2.3) [25]. This model allowed for both direct and indirect comparisons and ranking of treatments based on their relative effectiveness. Bayesian inference was performed using Markov Chain Monte Carlo (MCMC) simulation in MetaInsight (R v4.2.3). Four chains were run with 25,000 iterations each, discarding the first 5,000 as burn-in. A thinning interval of 1 was applied, yielding 20,000 posterior samples per chain (80,000 in total) for estimation of treatment effects and inconsistency via node-splitting. Local inconsistency within closed loops of the network was assessed using the node-splitting method. Convergence was assessed using Gelman-Rubin diagnostics (R̂ < 1.05) and inspection of trace plots. Model fit was evaluated using the deviance information criterion (DIC). Correlations in multi‑arm trials were modeled within the Bayesian framework to ensure appropriate weighting of arms.

Intervention relationships were visualized through network plots, and comparative effectiveness was ranked using surface under the cumulative ranking (SUCRA) probabilities, ranging from 0 (least effective) to 1 (most effective) [26]. A league table was constructed to present pairwise comparisons across all interventions for each outcome. To assess potential publication bias, comparison-adjusted funnel plots were generated using MetaAnalysisOnline (https://metaanalysisonline.com/netmetaeasy/) [27].

Sensitivity analyses were performed to evaluate the impact of study selection criteria on the robustness of the results. The initial network meta-analysis incorporated all eligible studies; however, to assess potential bias introduced by study quality, we systematically excluded trials identified as having a high risk of bias according to the RoB2 tool. Treatment effects were then re-estimated to determine whether the inclusion of lower-quality studies influenced the overall findings. Egger's regression test was applied to detect asymmetry, with results reported for both anxiety and heart rate outcomes.

Results

Study Selection

**Figure 1:**
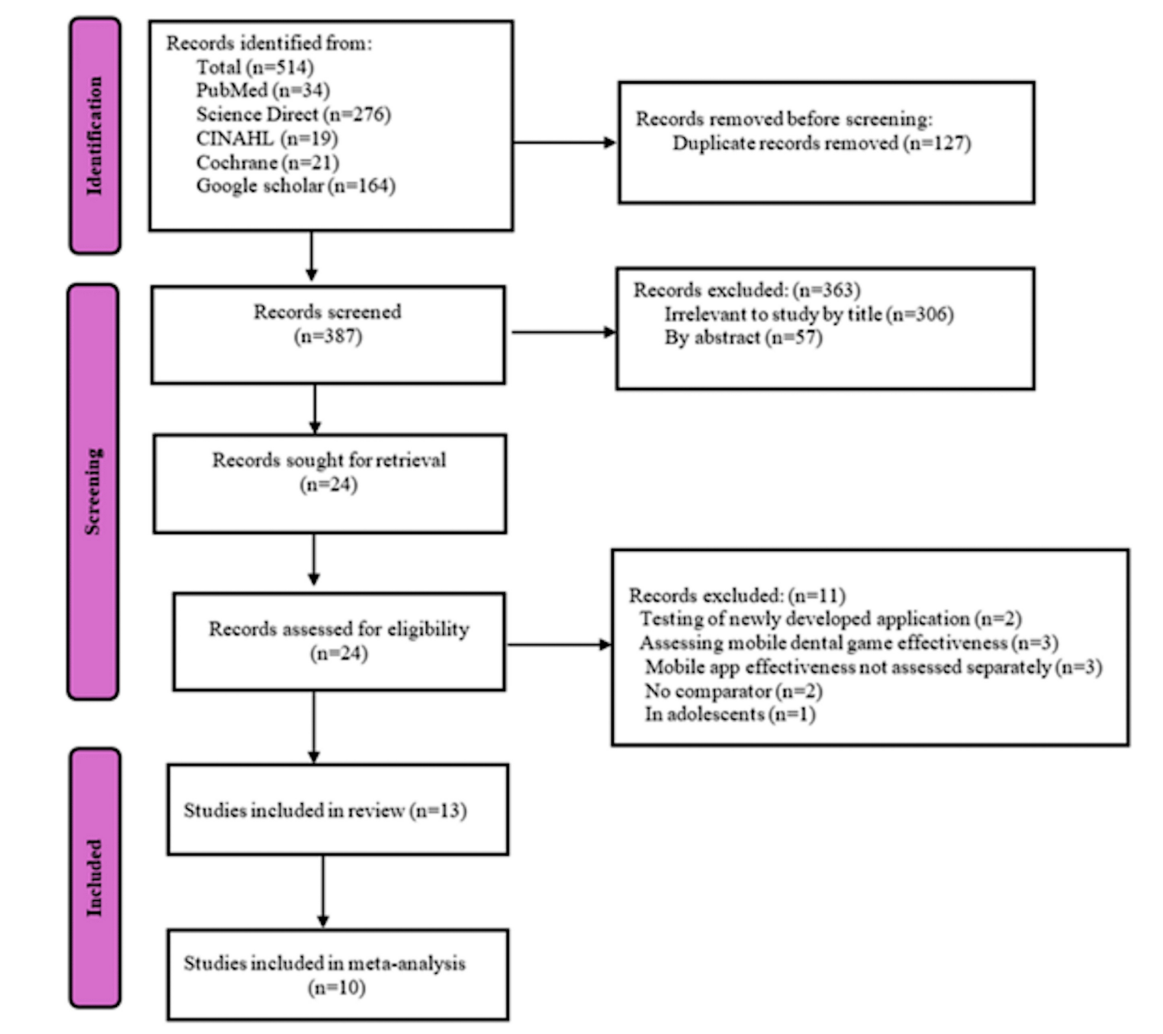
PRISMA chart (2020) This flowchart was created manually. PRISMA: Preferred Reporting Items for Systematic reviews and Meta-Analyses

A total of 514 records were initially identified through comprehensive database searches, including PubMed (n = 34), ScienceDirect (n = 276), CINAHL (n = 19), Cochrane Library (n = 21), and Google Scholar (n = 164). Following the removal of 127 duplicate entries, 387 records were screened based on titles and abstracts. Of these, 363 were excluded due to irrelevance, inappropriate study design, or insufficient information. Twenty-four full-text articles were retrieved and assessed for eligibility. Eleven studies were excluded at this stage for reasons including non-assessment of relevant outcomes, no comparator, testing of newly developed applications, and lack of separation of anxiety data in mixed-treatment populations. Ultimately, 13 studies met the inclusion criteria for qualitative synthesis [28-40], and 10 were eligible for quantitative meta-analysis [28,30,32-37,39,40]. The complete screening and selection process is illustrated in Figure 1.

Basic Characteristics of the Included Studies

A total of 12 RCTs and one non-randomized trial were included, conducted between 2019 and 2025 across six countries: India (n = 8) [28,29,32,33,36,37,39,40] Egypt (n = 2) [30,38], Iraq (n = 1) [34], Pakistan (n = 1) [31], and Syria (n = 1) [35]. The studies collectively enrolled 1,002 pediatric participants aged 4-11 years. Sample sizes ranged from 45 to 160, with balanced sex ratios reported in most trials.

Interventions included mobile dental applications (primarily game-based), animated videos, recorded maternal voice, and conventional behavioral techniques such as Tell-Show-Do (TSD), Tell-Play-Do (TPD), and video modeling. The mobile applications varied in design and developer origin, with most categorized as game-based educational tools. Dental procedures ranged from prophylaxis and oral screening to Class I restorations and pulpotomies. Outcomes assessed included physiological measures such as heart rate, pulse rate, and oxygen saturation (SpO₂), as well as behavioral and anxiety scales: Raghavendra, Madhuri, Sujata (RMS) Pictorial Scale, Venham's Picture Test (VPT), Facial Image Scale (FIS), Modified Child Dental Anxiety Scale (MCDAS-f), FLACC Scale, Frankl behavior rating, Chotta Bheem Chutki Scale (CBCS), Visual analog Scale (VAS), and Wright behavior rating scale. Across studies, mobile applications consistently demonstrated significant reductions in anxiety and behavioral distress, with some showing comparable or superior effects to conventional techniques. The detailed characteristics are presented in Table 3.

**Table 3:** Summary of findings of the included studies MP: Maternal presence; TPD: Tell-Play-Do; FIS: Facial image scale; TSD: Tell-Show-Do; VPI: Venham's Pictorial Index; CBCS: Chotta Bheem Chutki Scale; PR: Pulse rate; VAS: Visual analog scale; HR: Heart rate; NR: Not reported; RCT: Randomized controlled trial; RMS: Raghavendra, Madhuri, Sujata Pictorial Scale; MG: Mobile games

Author	Country	Study design	Mean age/range	Intervention groups	Sex ratio M:F	Sample size	Outcomes assessed	Name of mobile application (education/game)	Developer	Dental treatment provided	Findings
Elicherla SR et al., 2019 [28]	India	RCT	7–11 years	Mobile app, TSD	30:20	50 (n=25 per group)	PR, RMS Pictorial Scale	Little Lovely Dentist: Game-based educational app	Leaf Cottage Software and Shanghai Edaysoft Co., Ltd.	Dental prophylaxis	Significant anxiety reduction in the mobile app group (heart rate and RMS); TSD reduced RMS only
Kevadia M V et al., 2020 [29]	India	RCT	6–9 years	Film modeling, TPD, mobile app	NR	75 (n=25 per group)	HR, FIS, VPI	My Little Dentist: game-based educational app	Tenolgix Games	Oral screening, oral prophylaxis, and Class I restoration with GIC	The TPD group showed significantly lower HR, FIS, and VPI scores post-intervention and during treatment. While the “My Little Dentist” game delivered a clear subjective benefit in easing children’s self-rated dental anxiety, it was less effective at calming their physiological stress response compared to Tell-Play-Do. As a stand-alone modality, it can help reduce fear, but for maximal effect—especially on heart rate—TPD remains superior.
Derbala GA et al., 2021 [30]	Egypt	RCT	6–​​​​​​​​​​​​​​8 years	Mobile app, TSD	NR	76 (n=38 per group)	HR, VPI	Dentist Office Kids: Game-based educational app	NR	Class I restorations of carious maxillary primary molars under local anesthesia	The TPD group showed significantly greater reduction in anxiety than the TSD group
Abbasi et al., 2021 [31]	Pakistan	RCT	6–​​​​​​​​​​​​​​11 years	Mobile app, dental video, TSD, control	Mobile: 21:19; video: 19:21; TSD: 17:23; control: 22:18	160 (n=40 in each group)	HR, FIS	Little Lovely Dentist: Game-based educational app	Leaf Cottage Software and Shanghai Edaysoft Co., Ltd.	Dental prophylaxis	Mobile app and dental songs significantly reduced anxiety; tell-show-do increased it
Verma N et al., 2022 [32]	India	RCT	4.99 ± 0.92 years	TSD, TSD+MP, Mobile game (MG), MG+MP	50:30	80 (n=20 per group)	RMS-PS, FLACC scale, Frankl behavior rating scale	“Dentist”	YovoGames (age 4+)	Glass ionomer cement (GIC) restorations for primary second molars	All groups showed behavioral improvement. MG + maternal presence had the most favorable outcomes across all scales.
Panchal J et al., 2022 [33]	India	RCT	4–​​​​​​​​​​​​​​7 years	Recorded maternal voice via headphones, Roogies mobile app	40:40	80 (n=40 per group)	HR, VPI	Roogies: Cognitive-behavioral educational app	NR	Oral prophylaxis	Both interventions reduced anxiety, but the recorded maternal voice showed significantly greater reduction in heart rate and VPT scores during and after treatment
Aziz SZ et al., 2023 [34]	Iraq	RCT	6​​–8 years	TSD, mobile app, control	36:30	66(N=22 per group)	HR, SpO₂, VPT	Little Lovely Dentist: Game-based educational app	Leaf Cottage Software and Shanghai Edaysoft Co., Ltd.	Restorations of primary molars under local anesthesia	The mobile app group showed significantly lower heart rate and VPT scores, and higher SpO₂ levels post-treatment, compared to the TSD and control groups
Karkoutly M et al., 2024 [35]	Syria	RCT	7.47 ± 1.08 years	TSD, Mobile app	43:17	60 (n=30 per group)	PR, RMS Pictorial scale, FLACC behavioral pain scale	Baby Panda Dental Care: Game-based educational app	BabyBus Co., Fuzhou, China	Primary molars pulpotomy	The mobile game group showed significantly lower anxiety and pain
Nair SM et al., 2024 [36]	India	RCT	4–​​​​​​​​​​​​​​8 years	TSD, TSPD, Mobile app	37:23	60 (n=20 per group)	MCDAS-f, VAS, PR, SpO₂	Baby Shark – Dentist Game: Game-based educational app	Andre Scheidemantel	Class I restorations using Glass Ionomer Cement	TSPD was most effective in reducing anxiety (significant drop in MCDAS-f, VAS, pulse rate); the smartphone app was also effective but less than TSPD
Santhoshpriya AKR et al., 2024 [37]	India	RCT	4–​​​​​​​​​​​​​8 years	Mobile app, Dental video, TSD	NR	45 (n=15 per group)	PR, SpO₂, FIS, CBCS	Little Lovely Dentist: Game-based educational app	Leaf Cottage Software and Shanghai Edaysoft Co., Ltd.	Class I restorations using Glass Ionomer Cement	Significant anxiety reduction in mobile app and video groups (CBCS and FIS); physiological parameters showed no significant intergroup difference
Sedky MM et al., 2024 [38]	Egypt	RCT	5.0 ± 0.68 years (App group), 5.12 ± 0.79 years (TSD group)	Mobile app, TSD	44:36	80 (n=40 per group)	PR, RMS pictorial scale, VAS, Venham scale, Frankl scale	Little Lovely Dentist: Game-based educational app	Leaf Cottage Software and Shanghai Edaysoft Co., Ltd.	Local anesthesia followed by dental treatment	The mobile app group showed lower anxiety and pain scores and better cooperation
Chintala M et al., 2025 [39]	India	RCT	8.47 ± 2.18 years	Video modeling, Animated videos, Mobile app	54:36	90 (n=30 per group)	PR, RMS Pictorial scale	Little Lovely Dentist: Game-based educational app	Leaf Cottage Software and Shanghai Edaysoft Co., Ltd.	Noninvasive procedures: oral prophylaxis, restorations, pit and fissure sealants	All interventions reduced anxiety; Little Lovely Dentist showed the greatest reduction
Sasikumar T et al., 2025 [40]	India	RCT	69 years	Dentist bling app, Control	NR	100 (n=50 per group)	VPI, Faces anxiety rating scale, Wright behaviour rating scale	Dentist bling: Game-based educational app	Crazy Labs Ltd.	Prophylaxis and Class I restoration (mandibular arch)	Significant reduction in anxiety and improved cooperation in the app group; VPT and Faces scores dropped markedly post-intervention

Results of the Methodological Quality Assessment of the Included Studies

**Figure 2:**
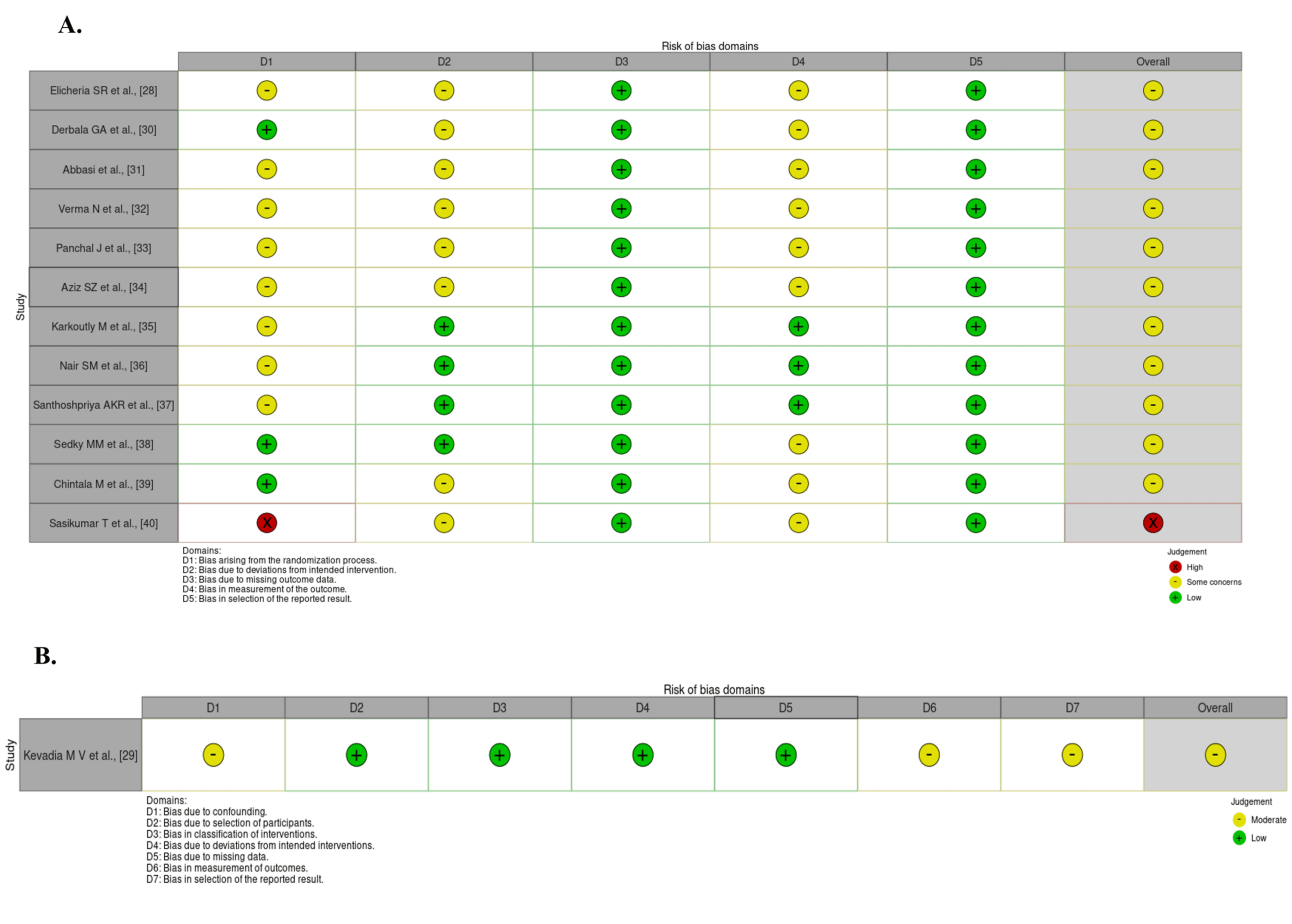
Risk of bias of the included studies: (A) randomized clinical trials; (B) non-randomized trials This is created using the RobVis tool (McGuinness LA & Higgins JPT, Bristol, United Kingdom; web-based platform).

The results of the risk of bias assessment conducted on the 12 included RCTs [28,30-40] and one non-randomized trial [29] are illustrated in Figure 2.

Regarding the randomization process, 11 studies were judged to have "some concerns," primarily due to insufficient detail on random sequence generation or lack of clarity around allocation concealment [28,31-37]. These limitations raised the possibility of baseline imbalances between intervention groups. Regarding deviations from the intended interventions, eight studies were rated as "some concerns," reflecting the inherent difficulty of implementing blinding in behavioral interventions involving mobile applications [28,30-34,39,40]. Most studies did not report whether participants or personnel were blinded to group allocation, which may have introduced performance bias.

For missing outcome data, all studies were rated as "low risk," as attrition rates were minimal and outcome data were consistently reported [28,30-40]. Similarly, in the domain of outcome measurement, nine studies were rated as "some concerns" due to a lack of blinding of outcome assessors or insufficient reporting on measurement procedures [28,30-34,38-40]. The remaining studies provided adequate detail and were rated as "low risk" [35-37]. In the domain of selective reporting, all studies were judged to be at low risk, with clearly defined outcomes and consistent reporting across study protocols and results sections [28,30-40].

Overall, 12 studies were rated as having some concerns in the risk of bias [28-39], and one study was rated as high risk [40]. Despite the presence of moderate concerns in several domains, particularly related to blinding and allocation concealment, the overall methodological quality of the included studies was considered acceptable for synthesis.

Network Analysis Results

**Figure 3:**
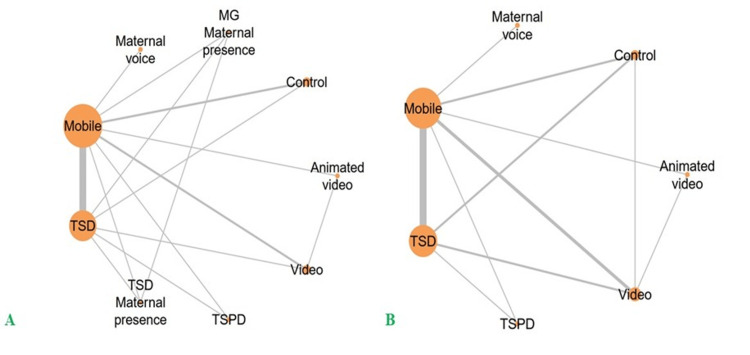
Network analysis: (A) dental anxiety; (B) heart rate This figure was generated by metaanalysisonline.com.

Network graph: This network meta-analysis synthesized data from 10 RCTs [28,30,32-37,39,40] evaluating nine distinct non-pharmacological strategies for managing pediatric DA. The interventions included TSD, TSPD, maternal voice, mobile application, animated video, standard video, control, TSD combined with maternal presence, and distraction via mobile games with maternal presence (MG + MP). The graphical representation of the network illustrates the comparative relationships among interventions for the DA outcome. In this diagram, line thickness corresponds to the number of studies contributing to each direct comparison, while node size reflects the total number of participants assigned to each intervention group (Figure 3). 

For DA, the network included 10 studies assessing nine interventions, with 707 participants contributing to 36 possible pairwise comparisons, of which 15 were supported by direct evidence. The network was fully connected, comprising both two-arm (n=5) and multi-arm (n=5) trials. Node-splitting analysis revealed no statistically significant differences between direct and indirect comparisons for video versus TSD (P=0.38995) or control versus TSD (P=0.491175). For heart rate, the network evaluating heart rate outcomes comprised nine studies investigating seven interventions, with a total of 687 participants. Of the 21 theoretically possible pairwise comparisons, 11 were supported by direct evidence. The network was fully connected, incorporating both two-arm (n=4) and multi-arm (n=5) trials. Node-splitting analysis revealed no statistically significant differences between direct and indirect comparisons for video versus TSD (P=0.131675) or control versus video (P=0.379575). In both outcomes, network estimates remained within the 95% credible intervals, indicating consistency between direct and indirect evidence sources and no notable divergence across comparisons. Furthermore, results from the sensitivity analysis were concordant with the main analysis, reinforcing the robustness of the treatment effect estimates. The estimated between‑study standard deviation was 0.77 (95% credible interval: 0.27-1.85), suggesting moderate heterogeneity across the network.

Outcome: Dental Anxiety

A total of 10 RCTs [28,30,32-37,39,40] assessed DA outcomes across a combined sample of 707 pediatric participants, evaluating the comparative effectiveness of nine distinct non-pharmacological interventions. The comparative analysis demonstrated that the TSPD technique was significantly more effective than all other interventions in reducing DA, yielding an SMD of −0.73 (95% CI: −1.76 to 0.30) when compared to mobile app-based approaches. Mobile apps also showed a statistically significant advantage over the TSD method (SMD = −0.89, 95% CI: −1.31 to −0.47). In contrast, comparisons with maternal voice (SMD = −0.55, 95% CI: −1.61 to 0.51), animated video (SMD = −0.56, 95% CI: −1.58 to 0.46), standard video (SMD = −0.70, 95% CI: −1.46 to 0.07), and the control group (SMD = −1.35, 95% CI: −2.08 to −0.62) did not reach statistical significance (Table 4).

**Table 4:** League table for dental anxiety assessment MP: Maternal presence; TSPD: Tell-Show-Play-Do; TSD: Tell-Show-Do; MG: Mobile games

TSPD								
-0.17 [-1.65; 1.31]	Maternal voice							
-0.64 [-2.06; 0.78]	-0.47 [-1.93; 1.00]	MG + MP						
-0.73 [-1.76; 0.30]	-0.55 [-1.61; 0.51]	-0.09 [-1.10; 0.93]	Mobile					
-0.85 [-2.27; 0.56]	-0.68 [-2.15; 0.79]	-0.21 [-1.36; 0.93]	-0.13 [-1.14; 0.89]	TSD + MP				
-1.29 [-2.73; 0.15]	-1.11 [-2.58; 0.36]	-0.65 [-2.08; 0.78]	-0.56 [-1.58; 0.46]	-0.43 [-1.86; 0.99]	Animated video			
-1.42 [-2.69; -0.15]	-1.25 [-2.56; 0.06]	-0.78 [-2.04; 0.47]	-0.70 [-1.46; 0.07]	-0.57 [-1.82; 0.68]	-0.14 [-1.15; 0.88]	Video		
-1.62 [-2.66; -0.58]	-1.45 [-2.59; -0.31]	-0.98 [-2.00; 0.04]	-0.89 [-1.31; -0.47]	-0.77 [-1.78; 0.25]	-0.33 [-1.41; 0.75]	-0.20 [-1.02; 0.63]	TSD	
-2.08 [-3.32; -0.83]	-1.90 [-3.19; -0.62]	-1.44 [-2.67; -0.21]	-1.35 [-2.08; -0.62]	-1.23 [-2.46; 0.00]	-0.79 [-2.04; 0.46]	-0.65 [-1.70; 0.39]	-0.46 [-1.24; 0.33]	Control

**Figure 4:**
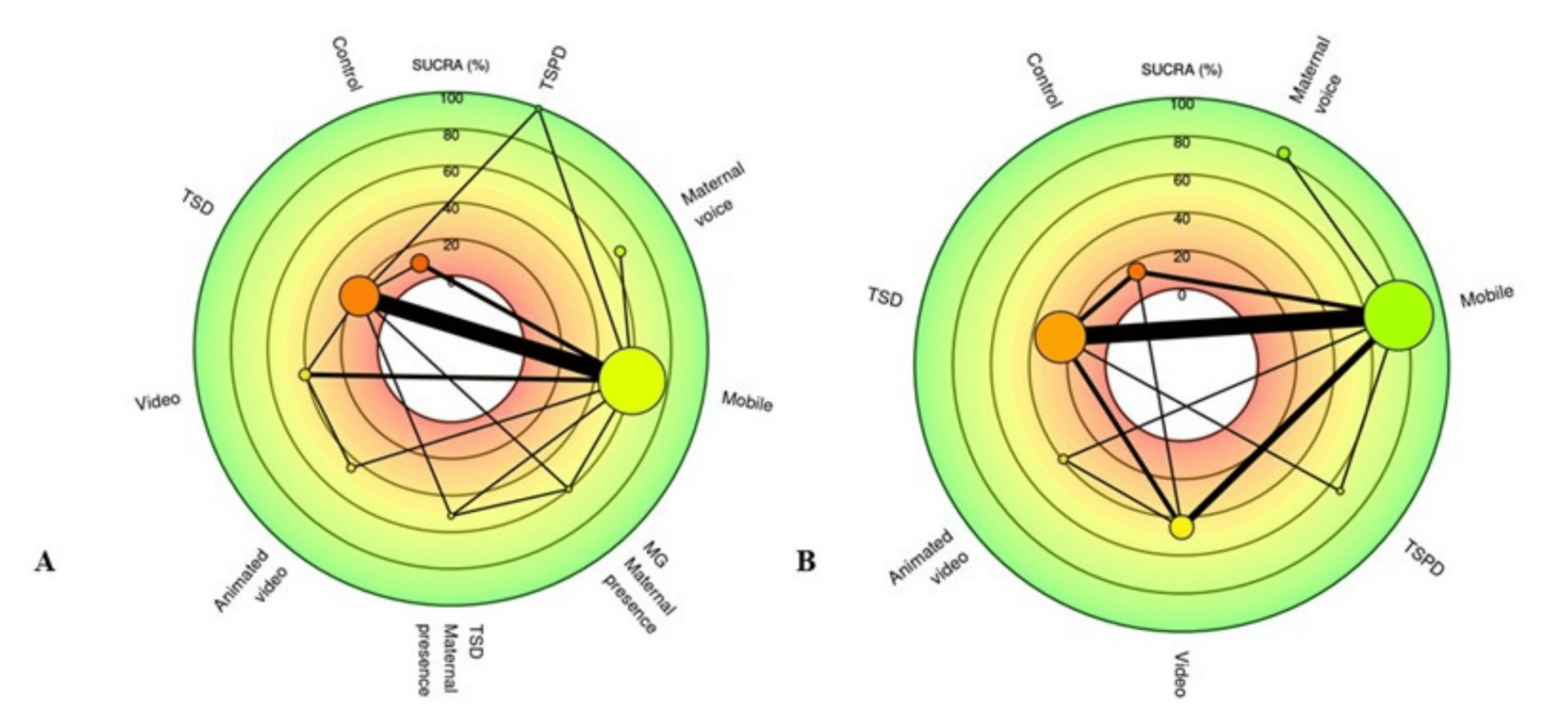
Comparative SUCRA rankings of pediatric anxiety management techniques. Color-coded concentric rings in the radar chart represent SUCRA efficacy tiers where green denotes high-ranking interventions (SUCRA >80%), yellow indicates moderate efficacy (SUCRA 50–80%), and red reflects low-ranking techniques (SUCRA <50%). Each node represents a distinct pediatric behavior guidance technique, plotted according to its SUCRA score. (A) Dental anxiety; (B) heart rate This figure was generated by metaanalysisonline.com.

Based on cumulative probability rankings, TSPD, maternal voice, and mobile apps emerged as the top three interventions for anxiety reduction, with SUCRA values of 99.53%, 66.34%, and 60.23%, respectively. The remaining interventions were ranked as follows: MG + MP (59.85%), TSD + maternal presence (51.11%), animated video (44.72%), standard video (40.88%), TSD (17.61%), and control (9.73%) (Table 5, Figure 4).

**Table 5:** SUCRA rank table for anxiety and heart rate SUCRA: Surface under the cumulative ranking curve; TSPD: Tell-Show-Play-Distraction; TSD: Tell-Show-Do; MG: Mobile games; HR: Heart rate

Treatment	Anxiety (%)	HR (%)
Animated video	44.72	39.36
Control	9.73	14.33
Maternal voice	66.34	83.24
MG Maternal presence	59.85	-
Mobile	60.23	76.5
TSD	17.61	25.13
TSD Maternal presence	51.11	-
TSPD	99.53	66.25
Video	40.88	45.18

Outcome: Heart Rate

A total of nine RCTs [28,30,33-37,39,40] assessed heart rate outcomes across a combined sample of 687 pediatric participants, evaluating the comparative effectiveness of seven distinct non-pharmacological interventions. The comparative analysis revealed that mobile app-based interventions were significantly more effective than the control group (SMD = −8.10, 95% CI: −13.30 to −2.89) and the TSD technique (SMD = −6.28, 95% CI: −10.00 to −2.56), indicating a substantial reduction in physiological anxiety. Comparisons with maternal voice (SMD = −2.35, 95% CI: −10.17 to 5.47), animated video (SMD = −4.88, 95% CI: −13.25 to 3.50), standard video (SMD = −3.90, 95% CI: −8.97 to 1.18), and the TSPD technique (SMD = −0.70, 95% CI: −8.60 to 7.20) also favored mobile apps, though these differences did not reach statistical significance due to wide confidence intervals (Table 6).

**Table 6:** League table for heart rate assessment MP: Maternal presence; TSPD: Tell-Show-Play-Do; TSD: Tell-Show-Do; MG: Mobile games

Maternal voice						
-2.35 [-10.17; 5.47]	Mobile					
-3.05 [-14.17; 8.07]	-0.70 [-8.60; 7.20]	TSPD				
-6.25 [-15.57; 3.08]	-3.90 [-8.97; 1.18]	-3.19 [-12.32; 5.94]	Video			
-7.23 [-18.69; 4.24]	-4.88 [-13.25; 3.50]	-4.17 [-15.60; 7.26]	-0.98 [-9.63; 7.67]	Animated video		
-8.63 [-17.29; 0.03]	-6.28 [-10.00; -2.56]	-5.58 [-13.53; 2.37]	-2.39 [-7.81; 3.04]	-1.41 [-10.34; 7.53]	TSD	
-10.45 [-19.84; -1.05]	-8.10 [-13.30; -2.89]	-7.40 [-16.53; 1.74]	-4.20 [-10.46; 2.06]	-3.22 [-12.80; 6.35]	-1.81 [-7.13; 3.50]	Control

Based on cumulative probability rankings, maternal voice, mobile apps, and TSPD emerged as the top three interventions for heart rate reduction, with SUCRA values of 83.24%, 76.5%, and 66.25%, respectively. The remaining interventions were ranked as follows: standard video (45.18%), animated video (39.36%), TSD (25.13%), and control (14.33%) (Table 5, Figure 4).

Publication Bias

**Figure 5:**
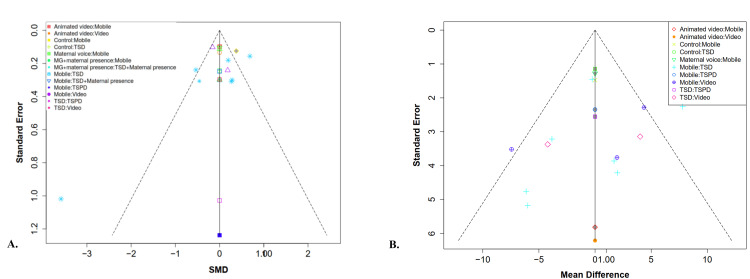
Funnel plot: (A) dental anxiety; (B) heart rate This figure was generated by metaanalysisonline.com.

Funnel plots were used to evaluate potential publication bias across all outcome indicators. Visual inspection of the funnel plots for DA and heart rate suggested asymmetry, indicating possible publication bias. This was further supported by Egger's regression test, which yielded statistically significant intercepts for both outcomes: for heart rate, the intercept was −15.52 (SE = 3.728), and for DA, the intercept was −1.75 (SE = 0.475). The funnel plots are presented in Figure 5.

Discussion

This network meta-analysis systematically compared the effectiveness of mobile oral health applications against conventional non-pharmacological behavioral techniques in reducing pediatric DA. By synthesizing data from 10 RCTs involving 707 children aged 4-11 years, this study provides a comprehensive framework for evaluating digital interventions, maternal voice strategies, and traditional chairside techniques such as TSD and TSPD.

This study findings indicate that the TSPD technique demonstrated the highest efficacy in reducing DA, followed closely by recorded maternal voice and mobile applications. These results align with prior meta-analyses highlighting the importance of multisensory engagement and parental presence in managing anxiety during pediatric dental procedures [41]. The TSPD technique integrates visual, auditory, and tactile stimuli, creating a playful and immersive environment that diverts attention away from fear-inducing stimuli. This approach fosters a sense of familiarity and control, making it particularly effective in younger children whose cognitive development favors sensory-based learning and distraction [32].

Mobile oral health applications, especially those with game-based platforms such as "Little Lovely Dentist" and "Baby Panda Dental Care," were found to significantly reduce anxiety scores compared with traditional methods. These applications simulate dental environments using interactive storytelling, animated characters, and procedural modeling, allowing children to experience dental visits in a non-threatening context. The gamified structure encourages children to participate in virtual dental tasks such as cleaning teeth and applying fillings, which promotes procedural familiarity and reduces anticipatory anxiety. The use of reward-based reinforcement in these apps also enhances behavioral compliance and emotional regulation, supporting their utility in pediatric settings [37,38].

From a physiological perspective, mobile interventions were associated with reductions in heart rate and improved oxygen saturation, indicating a dampening of sympathetic nervous system activity during treatment. This autonomic modulation may be attributed to the cognitive distraction and emotional engagement provided by the apps, which shift the child’s focus away from perceived threats and toward goal-directed play. These findings support the notion that digital interventions not only reduce subjective anxiety but also positively affect physiological stress responses [42].

However, the effectiveness of mobile apps appeared to be influenced by the presence of maternal support. Studies incorporating maternal voice or maternal presence alongside digital tools reported better outcomes, suggesting a synergistic effect between emotional reassurance and cognitive distraction. This is consistent with developmental psychology literature that underscores the role of parental cues in modulating stress responses in young children, further emphasizing the importance of combining emotional support with technological interventions for optimal anxiety reduction [43].

Chairside techniques like TSD and TPD, while integral to pediatric dentistry, demonstrated comparatively lower efficacy in this analysis. These techniques rely heavily on verbal explanation and physical demonstration, which may be less engaging for children who are accustomed to digital media. Moreover, the effectiveness of these methods is influenced by the clinician’s communication style, the child’s developmental stage, and the clinical environment. While still valuable, these techniques could be enhanced by incorporating digital tools or parental support strategies to maximize their impact on reducing anxiety [38].

Animated dental videos, which visually model procedures in a non-threatening format, showed moderate anxiety reduction. These visual aids, often in cartoon or simulation style, help familiarize children with dental procedures through narrative modeling and visual desensitization. However, standalone animated videos were less effective in reducing anxiety scores compared to interactive mobile applications or caregiver-assisted approaches [44]. As such, these videos are best utilized as adjuncts to more interactive, multimodal behavior guidance strategies.

The study highlights the evolving landscape of pediatric anxiety management, where traditional behavior guidance is increasingly complemented by technology-driven and emotionally supportive interventions. The integration of mobile applications into clinical workflows offers a scalable, non-invasive, and child-friendly approach to improving treatment experiences and reducing behavioral resistance.

Since there is moderate heterogeneity in the network, the SUCRA rankings should be interpreted with caution. Although SUCRA provides a probabilistic hierarchy of interventions, variability across studies implies that apparent differences in ranking may not represent clinically meaningful distinctions. This uncertainty highlights the importance of contextualizing treatment hierarchies within the broader evidence base and underscores the need for further high‑quality randomized trials to confirm the robustness of these comparative findings.

Limitations and Future Research

Despite promising results, several limitations must be acknowledged. The heterogeneity in app design, developer origin, and content delivery introduces variability in intervention fidelity. Likewise, included studies varied in their use of anxiety scales, ranging from the RMS Pictorial Scale to MCDAS-f and CBCS, which may affect cross-study comparability. While younger children appeared more responsive to animated and game-based interventions, further age-specific trials are needed to confirm these trends. Additionally, cultural factors and language localization of mobile apps may influence engagement and efficacy, warranting region-specific validation.

## Conclusions

TSPD ranked highest for pediatric DA reduction, followed by maternal voice interventions and mobile oral health applications. These approaches significantly outperformed other nonpharmacological behavior guidance techniques, with maternal voice and mobile platforms also showing strong effects on physiological outcomes such as heart rate. Collectively, the evidence supports multimodal, child‑centered strategies, particularly those integrating digital engagement with caregiver involvement as optimal complements to conventional methods. However, these findings should be interpreted with caution, as the network meta‑analysis demonstrated moderate heterogeneity across included studies.
